# Sunflower sea star chemical cues locally reduce kelp consumption by eliciting a flee response in red sea urchins

**DOI:** 10.1098/rspb.2025.0949

**Published:** 2025-07-09

**Authors:** Raphael T. Mancuso, Sarah A. Gravem, Rose S. Campbell, Nathan Hunter, Pete Raimondi, Aaron W. E. Galloway, Kristy J. Kroeker

**Affiliations:** ^1^ Department of Ecology and Evolutionary Biology, University of California Santa Cruz, Santa Cruz, CA, USA; ^2^ Oregon State University, Corvallis, OR, USA; ^3^ Oregon Institute of Marine Biology, University of Oregon, Eugene, OR, USA

**Keywords:** sea star wasting disease (SSWD), top-down control, behaviourally mediated trophic cascade, landscape of fear, kelp restoration, trophic connections

## Abstract

Predator loss can cause shifts in ecosystem state, especially when accompanied by changes in the behaviour of its prey. The recent decline of predatory sunflower sea stars *Pycnopodia helianthoides* and a coincident a decline of kelp forests across the northeastern Pacific raises questions about their role in kelp forest ecosystem maintenance and recovery. While *P. helianthoides* may support kelp forests by consuming herbivorous sea urchins, less is known about their non-consumptive effects, especially in degraded habitats. Here, we ask: How do the non-consumptive effects of this predator vary among prey species, and what are the emergent effects on community-level grazing pressure in a degraded habitat when kelp is reintroduced? We conducted a field experiment, where we deployed kelp blades in urchin barrens at discrete distances from caged *P. helianthoides* and control cages for 24 h. We demonstrate a reduction in the density of red sea urchins *Mesocentrotus franciscanus* nearest the sunflower star and no effect on green sea urchins *Strongylocentrotus droebachiensis*. Our results suggest the chemical cue of *P. helianthoides* elicits a localized landscape of fear of approximately 15 m^2^ that suppresses grazing, and that the non-consumptive effects of *P. helianthoides* on sea urchin behaviour may be important for kelp restoration.

## 1. Introduction

Predators can play an important role in ecosystem recovery and restoration success by re-establishing trophic cascades that facilitate recovery of habitat-forming species [1,2]. Behaviourally mediated trophic cascades, where the risk of predation influences prey traits with subsequent effects on lower trophic levels [3,4], may be especially important for restoration efforts because a reintroduced predator has the potential to impact many more prey individuals via changes in their behaviour/traits than via direct consumption. In practice, the determinants of the strength of behaviourally mediated trophic cascades are poorly understood, especially in complex, functioning ecosystems with inherent variability and biodiversity [5–8]. For example, reintroduced predators in complex food webs often prey on multiple species. In ecosystems with multiple prey, the strength of a behaviourally mediated trophic cascade will depend on the predator’s non-consumptive effects on each prey species [9], the interactions among prey species (e.g. interference competition), as well as the interaction strength of each prey species on the lower trophic levels. Moreover, in restoration settings where the prey species have already degraded the lower trophic level, the energetic status of the prey (e.g. hunger level) can weaken the strength of the behaviourally mediated trophic cascade if a prey species is willing to take greater predation risks to acquire food [10,11]. Finally, the density of prey species, which can be high in degraded ecosystems, can also influence risk assessment and behaviour [12,13]. Therefore, quantifying the variability in the non-consumptive effects of a predator on multiple prey, as well as the emergent effects on lower trophic levels in degraded ecosystems, is crucial for understanding their potential use in restoration.

Kelp (Order Laminariales) forests are highly productive, economically important and culturally valued ecosystems that occur across the temperate and arctic regions of the world’s oceans [14]. In recent years, kelp forests across large regions of the northeastern Pacific have undergone dramatic declines in kelp cover [15–18]. These declines have initiated intense interest in restoration strategies, including predator reintroductions [19,20]. Although there is variability in the relative strength of the top-down control of kelp forest community structure across the region, kelp forests in the northeastern Pacific are in part governed by trophic cascades [21–23]. When present, sea otters (*Enhydra lutris*), predatory fish (*Semicossyphus pulcher*) or lobsters (*Panulirus interruptus*) can indirectly maintain kelp biomass by consuming herbivorous sea urchins, thereby controlling shifts between stable states of highly productive kelp forests and vegetatively desolate ‘urchin barrens’ [21,24–26]. Urchin barrens are defined by high densities of sea urchins lacking gonads and the absence of habitat forming kelp species. The recolonization of otters after their extirpation during the fur trade highlights the potential for predator reintroductions to facilitate the recovery of kelp biomass [25,27,28]. Kelp forest habitats with higher biomass of predatory fish and crustaceans have been shown to preserve kelp biomass as well [26]. The recent kelp forest declines in the northeastern Pacific were most pronounced in areas where these top predators like sea otters were already absent or were limited/patchy in their distribution [15,17,18,29], suggesting trophic cascades may have played some role in the ecosystem collapse.

The sunflower sea star (*P. helianthoides*) is one predator in this system whose trophic interactions have garnered renewed attention for their potential role in kelp forest recovery and restoration. In 2013, a widespread marine disease referred to as sea star wasting disease (SSWD) caused sudden local extinctions of many sea star species across the northeastern Pacific [30,31]. *Pycnopodia* populations declined precipitously, with an estimated mortality of >90% throughout its range [32]. The aforementioned regime shifts towards urchin barrens followed this dramatic loss of *Pycnopodia* (and a concurrent marine heatwave) and were most dramatic in places where *Pycnopodia* were extirpated and other major urchin predators were rare or absent [15–18,33]. While it is widely expected that the *per capita* consumptive pressure of *Pycnopodia* is considerably less than an *E. lutris* that targets sea urchins, sea otters that prey on urchins can sometimes avoid the less calorically valuable individuals usually found in barrens [34] and focus their efforts elsewhere when better foraging options are available [29]. As generalist predators with diverse diets, labratory studies suggest *Pycnopodia* will prey on gonad-deficient urchins [35], which could strengthen the importance of their consumptive effects in kelp forest resilience and restoration success.

As a highly mobile, generalist predator, *Pycnopodia* may also exert strong non-consumptive effects on its prey via a waterborne chemical cue [36], but much is unknown about the cascading indirect effects of *Pycnopodia* on kelp recovery in degraded ecosystems. Several laboratory studies have demonstrated that proximity to *Pycnopodia* elicits an escape or defence response in sea urchins [37–39]. Moreover, purple sea urchins (*Strongylocentrotus purpuratus*), small red sea urchins (*Mesocentrotus franciscanus*), and green sea urchins (*S. droebachiensis*) consume less kelp in the presence of *Pycnopodia*’s chemical cue [36,37,40], suggesting its non-consumptive effects could trigger behaviourally mediated trophic cascades across multiple species. The footprint of a sea star’s non-consumptive effect on the grazer community may be especially large considering the sea star’s mobility and the dispersion of a cue in seawater. In contrast, the effects of a sea star on surrounding prey could be relatively small if the cue becomes too dispersed in naturally dynamic field settings. Moreover, given *Pycnopodia* is a generalist predator, it is unclear how these effects may differ among species or size classes of prey in the field. It has been hypothesized that large red sea urchins (*M. franciscanus*), capable of imparting significant grazing pressure, may have a size refuge from *Pycnopodia* predation [15,36]. If these larger individuals do not exhibit behavioural effects [37], then a behaviourally mediated trophic cascade would be constrained. Similarly, the non-consumptive effects of sea stars may differ among individuals based on their nutritional state [40], with hungry sea urchins in degraded habitats being less risk averse than their well-fed counterparts, particularly when food is available (e.g. kelp is reintroduced or starts to recover). Finally, the non-consumptive effects of *Pycnopodia* on different prey species may be influenced by the presence of other predators [24] or the density of conspecifics [41], which can be quite high in degraded urchin barrens.

In the past four decades, few studies have explored the non-consumptive effects of *Pycnopodia* on prey species and the cascading effects on primary producers in the field (but see [36,42]). Here, we attempt to fill this knowledge gap by experimentally manipulating the presence of *Pycnopodia* in urchin barrens to investigate its non-consumptive effects on the two dominant sea urchin species in southeast Alaska. In this study, we ask: What are the spatial and temporal effects of *Pycnopodia* presence on the behaviour of multiple sea urchin species and on community-level grazing pressure in urchin barrens when kelp is reintroduced to the degraded habitat? To answer this question, we conducted a field experiment in three replicate urchin barrens in Sitka Sound, Alaska. We deployed kelp blades at increasing distances around a caged adult *Pycnopodia* for 24 h. We assessed changes in the density of the two most numerically dominant herbivores, *M. franciscanus* and *S. droebachiensis*, as well as changes in community-level grazing pressure on the experimental kelp blades. Using changes in the sea urchin density and grazing pressure, we calculated the spatial scale of an individual sea star’s effect. Based on our initial observations, we predicted sea urchins would flee in response to *Pycnopodia*, and thus we expected to see initial reductions in sea urchin densities nearest to the sea star. We also hypothesized that the emergent effects on kelp after 24 h would be mediated by differences in the risk assessment of the prey species, who would be attracted to the kelp, with unknown effects on community-level grazing pressure.

## 2. Methods

### (a) Site characterization

We conducted our field experiment along the western coast of Baranof Island in Sitka Sound near the city of Sitka, Alaska. We performed our experiments at three sites approximately 6 km east of Sitka: Ellsworth Cut, Harris Island and Whale Park (table 1). These sites have low abundance of sea otters [16,43]. Furthermore, *P. helianthoides* have been either absent or very rare at these sites since the SSWD epidemic in 2013 [16] (K.J.K., personal observation). These three sites have been known to support kelp forest ecosystems in the last decade. As current urchin barrens that recently supported kelp communities, these sites are thus relevant to questions regarding the constraints on kelp forest ecosystem recovery [16,44].

**Table 1.** Characterizations of the three experimental urchin barren sites, as well as a reference kelp forest site (Magic Island). Grazer densities were gathered from a single swath transect survey at each site (60 m^2^ for Ellsworth and Harris and 30 m^2^ for Whale Park) and gonad indices were calculated for red urchins only (*n* = 11, 9, 10, 10 for each site, in order) as: (wet mass of gonadal tissue/total mass of the organism) × 100. Values are mean ± s.d.; ‘nd’ indicates no data.

site	ecosystem state	latitude	longitude	red urchin density (m^−2^)	green urchin density (m^−2^)	red urchin gonad index	red urchin mean test size (mm)	green urchin mean test size (mm)
Ellsworth Cut	urchin barren	57.036	−135.280	2.97	7.42	2.32 ± 2.23	44.83 ± 13.24	30.95 ± 11.12
Harris Island	urchin barren	57.033	−135.277	2.10	5.22	1.15 ± 0.86	48.80 ± 17.33	28.15 ± 7.58
Whale Park	urchin barren	57.033	−135.255	2.13	2.97	1.51 ± 1.28	41.09 ± 13.23	32.08 ± 15.7
Magic Island	kelp forest	57.098	−135.401	nd	nd	7.40 ± 3.08	nd	nd

The sites were qualitatively similar in rugosity (low rugosity), substrate (bedrock, with some boulder and cobbles) and depth (6−9 m). To test if these sites could be characterized as sea urchin barrens, we (i) surveyed the density and size structure of urchin species at each site and (ii) compared the gonad indices of red sea urchins collected at our three sites to an intact kelp forest reference site (Magic Island) about 12 km away from the nearest experimental site (table 1). Because the gonad indices were meant to provide corroboration for the observable pattern in ecosystem state, we did not collect gonads for both species. For the site characterization surveys, we quantified the densities of *M. franciscanus* and *S. droebachiensis* using a swath approach on SCUBA (2 × 30 m^2^ at Ellsworth Cut and Harris Island, and 2 × 15 m^2^ at Whale Point). We measured the gonad index of *M. franciscanus* individuals from each site (*n* = 9–11) as: [wet mass of gonadal tissue/total mass of the organism] × 100.

### (b) Cage experiment

**Figure 1.**
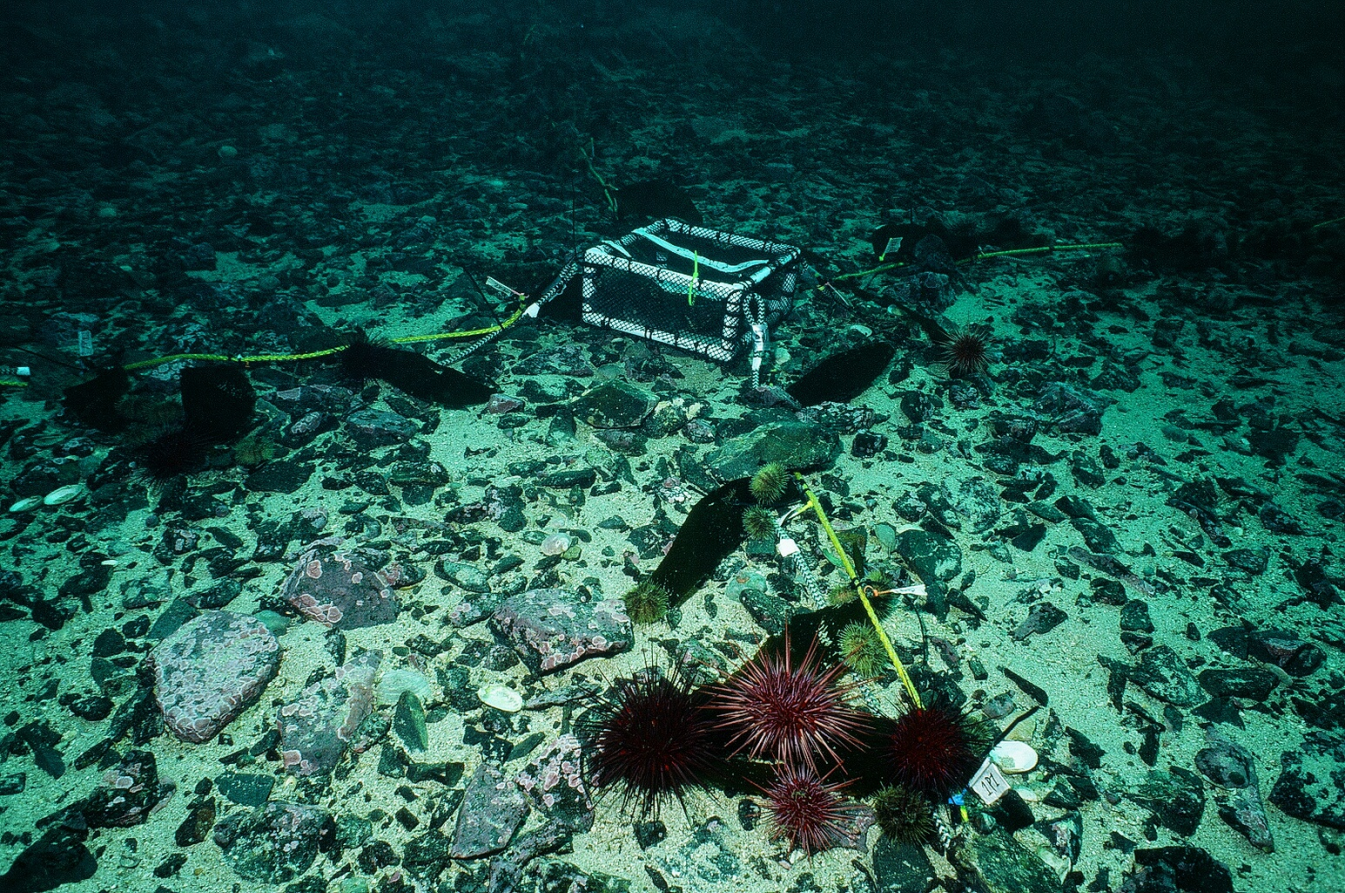
Photograph of an experimental array, showing the central cage housing a *Pycnopodia* with four 4 m long radial transects at each corner and attached kelp lines. At predetermined intervals along each transect, the density of grazers was surveyed before, just after (within 30 min), and 24 h after *Pycnopodia* and kelp lines were deployed. Just before the start of each trial, kelp blades of known weight were attached at predetermined intervals along each transect and grazing was allowed for 24 h before collection and re-weighing. The experiment started when a single *Pycnopodia* either was or was not added to the cage. Photo by R.T.M.

To test whether and at what distance the presence of *Pycnopodia* can reduce prey densities or suppress grazing on kelp, we performed an underwater caging experiment at each barren site. Each experimental array consisted of a central cage with four 4 m long radial transect lines (figure 1). We constructed cages (30 × 30 × 15 cm^3^; *l* × *w* × *h*) using a PVC frame covered in approximately 1 cm Vexar mesh fastened with zip ties that could be opened underwater to add a sea star. We attached a 4 m long lead line to each corner of the cage, forming a plus pattern (figure 1). We simultaneously deployed four of these arrays at each site in two blocks. In each block, one cage served as an experimental treatment (with a *Pycnopodia*) and the second cage served as a control (a cage without a *Pycnopodia*). In February 2023, we placed all arrays in areas with high sea urchin density, hard rocky substrate and low rugosity. The cages within a block were approximately 20–30 m apart.

Once the four cages and attached lead lines were deployed, but before adding kelp lines or sea stars to the treatment cages, we surveyed the initial densities of red and green sea urchins using 0.25 m^2^ quadrats at metre marks 0, 0.5, 1, 2 and 3.5 along each lead line. To each lead line of all cages, we attached a yellow nylon ‘kelp line’ with 1 m distances demarcated by tape (figure 1). Kelp lines consisted of *Macrocystis pyrifera* blades of known wet weight at regular intervals (metre marks: 0, 0.25, 0.5, 0.75, 1, 1.5, 2, 2.75, 3.75) woven into the nylon line. The experiment began when we sealed a *Pycnopodia* (9.5−19.5 cm radius, laboratory acclimated for >2 weeks during which the seastars were fed red urchins ad libitum) into the experimental treatment cage and the control cage was sealed with a dive weight. Between 15 and 30 minutes after sea stars and kelp lines were deployed (hereafter referred to as 0.5 h), we resurveyed the grazer densities in the same quadrat locations when possible (these surveys were sometimes limited by air availability on SCUBA). This resulted in nine surveys of density at 0.5 h. We then left the array overnight and surveyed grazer densities again at approximately 24 h (12 surveys). After the 24 h surveys, we removed the arrays and kelp lines and re-weighed each individual kelp blade in the labratory to calculate any change in wet mass.

### (c) Analyses

#### (i) Sea urchin densities

We tested the main and interactive effects of *Pycnopodia* treatment and the distance from the cage on the density of grazers in each quadrat using a generalized linear mixed effects model (tweedie distribution, log-link function), with *treatment* (*Pycnopodia* versus control), *time* (before, 0.5 and 24 h), and *species* (red versus green urchins) as categorical fixed effects, log_10_ *distance* from cage as a continuous fixed effect, and *site*, *block* and *transect* as random effects. We log-transformed the distance from the cage to account for the nonlinearity in the relationship between urchin densities and distance from the cage based on visual analysis. For random effects, we included *site*, *block* (nested within *site*) and *transect* (nested within *treatment*, which was nested within *block*, which was nested within *site*) to account for the non-independence of transects within an array, and blocks within a given site. Based on a visual analysis of normal–normal (*Q*–*Q*) plots and residuals, we used a tweedie distribution for the density data, and fitted the model with the glmmTMB package in R [45]. To disentangle significant three-way interactions in the model, we performed follow-up comparisons using the emmeans package in R [46], using emtrends to test for differences in the slopes of urchin density with distance from cage among species and different time points. Values of *p* from emmeans() and emtrends() are reported in the text for specific comparisons. Figures include the fitted model overlaid on the raw data on non-log-transformed axes (to show the nonlinear relationship).

To describe the changes in the urchin density gradient in the text, we also calculated the average urchin density close to (0 m) versus far from (3.5 m for urchins) the cage at 30 min and 24 h for each treatment, then calculated the % change in density with distance from the cage as (% change = ((density at 0 m − density at 3.5 m)/density at 0 m) × 100).

#### (ii) Kelp grazed

We also assessed the main and interactive effects of *Pycnopodia* treatment and the distance from the cage on the proportion of kelp biomass eaten using a general linear mixed effects model in R like above, with *treatment* (*Pycnopodia* versus control) and *time* (before, 0.5 and 24 h) as categorical fixed effects, log_10_ *distance* from cage as a continuous fixed effect, and *site*, *block* and *transect* as random effects. We again nested *block* within *site*, and *transect* within *treatment*, which was nested in *block* and *site*, as random factors. Based on visual analysis of the normal–normal (*Q*–*Q*) plots and residuals, we used lmerTest in package lmer [47] to fit the model for a normal distribution.

We then calculated the net effect of the sea star at 24 h on both red and green sea urchin abundance and kelp mass as the difference in the average urchin count or per cent of kelp remaining at a given distance on an array, respectively, in the sea star treatment minus its paired control treatment (i.e. the differences between treatments in each block at a given distance, after taking the mean of the four transects on an array). In other words, for each block and at a given distance from the cage at 24 hours we calculated: (avg. density of a given sea urchin species or per cent remaining in kelp in sea star treatments at each distance − avg. density or per cent remaining in paired control treatments at each distance). We tested the main and interactive effects of log_10_ *distance* from the cage and *species* (kelp, red or green urchins) on the net sea star effect using a linear model (lm) in base R. Finally, we calculated the radius of the ‘halo of influence’ of the sea star by setting the net effect to zero in the model equation for each species and solving for the distance to the cage (i.e. the distance at which the sea star effect was no longer detectable). We calculated the area of influence using *π* × (radius^2^), with the radius being defined as the halo of influence statistic solved from the previous equation. This gives us an approximate measure of how far from an inactive sea star we can expect sea urchin density and grazing to be suppressed for at least 24 h, even when kelp is present in an urchin barren.

## 3. Results

### (a) Site characterization

Across the three experimental sites, *S. droebachiensis* density was greater than *M. franciscanus* density (table 1), whereas *M. franciscanus* test sizes, on average, were larger than *S. droebachiensis* (table 1). The gonad index for *M. franciscanus* at each experimental urchin barren site was lower than that at the nearby kelp forest reference site (table 1), as expected.

### (b) Cage experiment

#### (i) Sea urchin densities

**Figure 2.**
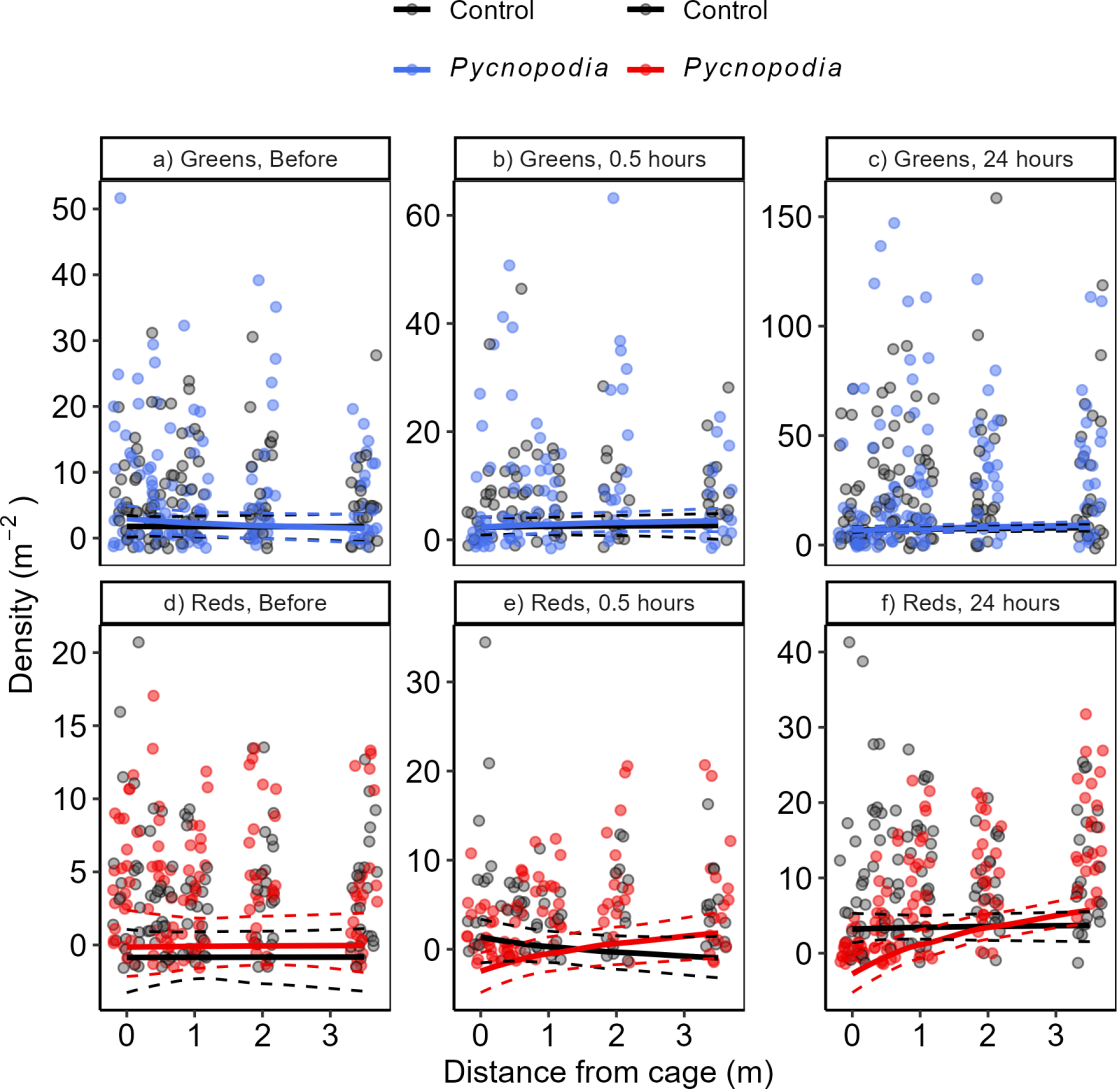
Densities of green (a–c) or red (d–f) sea urchins with untransformed distance from the cage containing either none (grey) or one (colours) *P. helianthoides* sea star. Lines are plotted as the generalized linear mixed model fit of density with the predicted fixed effects treatment (*Pycnopodia* versus cage control) with log_10_ *distance* from cage, overlaid on raw data. Dashed lines indicate 95% confidence intervals of model fits. Time points are before *Pycnopodia* and kelp deployment (a,d), 0.5 h after deployment (b,e) or 24 h after deployment (c,f). Red and green urchins were *M. franciscanus* and *S. droebachiensis*, respectively. Differences in the number of data points at 0.5 h is due to fewer surveys at this time point. Note different *y*-axis scales.

The effects of *Pycnopodia* (*treatment*) on sea urchin density as a function of the distance from the cage differed among time points and species (i.e. three-way interactions between *treatment ×* log_10_ *distance* and *species*, as well as *time point*; table 2). Within 30 min of the experiment starting (start is when *Pycnopodia* and kelp lines were deployed) and after 24 h, there is no clear change in the density of green urchins as a function of *Pycnopodia* treatment (figure 2a–c; *p* = 0.999 for both 0.5 and 24 h). However, green urchins were attracted to the kelp, and overall green urchin density increased by 293% in both sea star treatments and controls combined by 24 h due to immigration (*p* < 0.001).

**Table 2.** Results of a generalized linear mixed effects model testing the main and interactive effects of sea star treatment, log_10_ *distance* from cage, time point, and species on the abundance of red and green sea urchins. Models included *site*, *block* (nested within site) and *transect* (nested within site, block and treatment) as random factors.

effect	Chisq	d.f.	**Pr(>Chisq)**
treatment	0.14	1	0.711
log distance	32.74	1	<0.001
time point	541.28	2	<0.001
species	320.60	1	<0.001
treatment : log distance	17.54	1	<0.001
treatment : time point	3.93	2	0.140
log distance : time point	27.11	2	<0.001
treatment : species	6.04	1	0.014
log distance : species	5.32	1	0.021
time point : species	29.61	2	<0.001
treatment : log distance : time point	12.49	2	0.002
treatment : log distance : species	17.16	1	<0.001
treatment : time point : species	6.26	2	0.044
log distance : time point : species	2.28	2	0.320
treatment : log distance : time point : species	2.86	2	0.240

In contrast, red urchins moved away from the caged sea stars, but closer to the empty cages in the control treatments within 30 min (figure 2d–e; *p* = 0.002). In sea star treatments, the density of red urchins was 187.5% lower near versus far from the sea star after 30 min (figure 2e). In kelp only treatments (controls), the density of red urchins was 48.3% higher near the cage than far from the cage, suggesting red urchins were attracted to cages with kelp alone. These patterns appeared to be caused primarily by movement of red urchins within the arrays rather than immigration or emigration, because the overall density in the arrays remained similar within the first 30 min regardless of treatment (figure 2e; *p* = 0.097). After 24 h, the antipredator response by red urchins intensified, with 3066% fewer red sea urchins close to the sea stars than far from the sea stars (figure 2f; *p* < 0.001). While the attraction to the control cage itself noted at 30 min disappeared by 24 h (i.e. the *treatment ×* log_10_ *distance* effect; figure 2e–f), we found that red urchins were attracted to the kelp arrays without sea stars, and their density increased overall by 193% after 24 h (i.e. a *treatment* effect for reds at 24 h; *p* < 0.001). Sea stars suppressed this immigration such that there was only an 87.5% increase in red urchins to kelp arrays when sea stars were present (*p* = 0.382).

#### (ii) Kelp grazed

**Figure 3.**
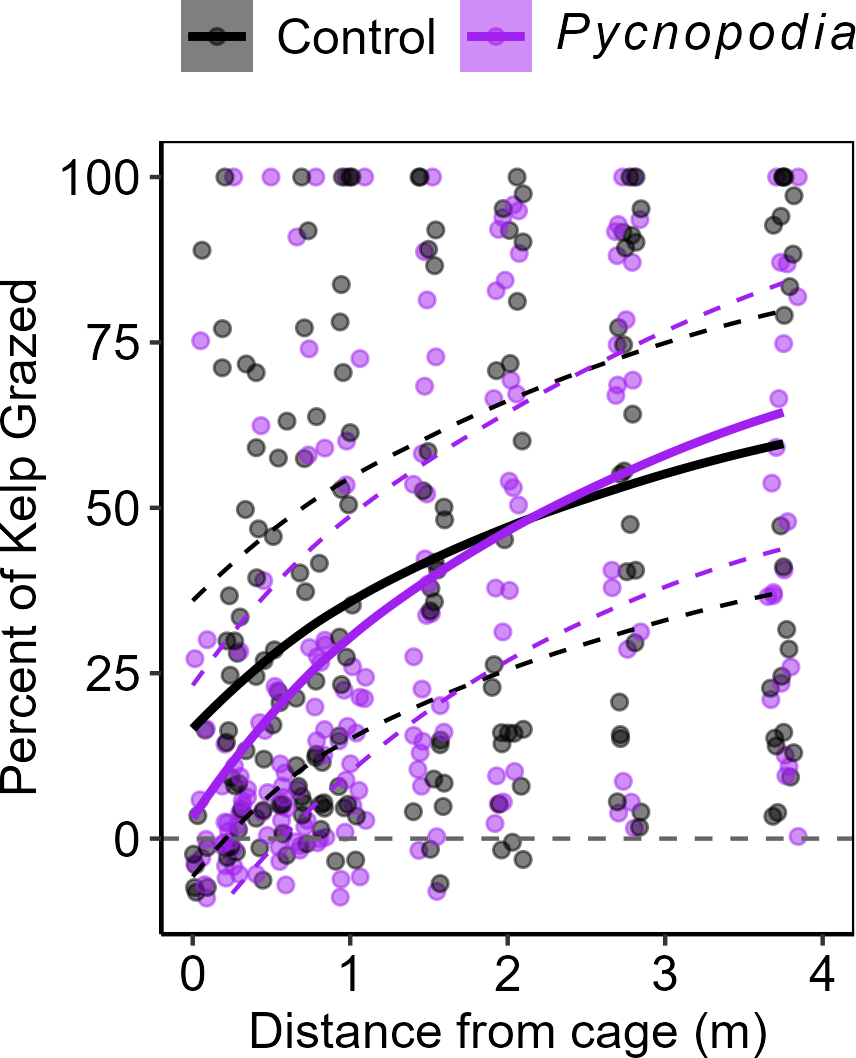
Percentage of *Macrocystis pyrifera* kelp grazed after 24 h with untransformed distance from the cage containing either none (grey) or one (purple) *Pycnopodia helianthoides* sea star. Lines are plotted as the general linear model fits of per cent of kelp grazed with the predicted fixed effects of *Pycnopodia* and cage control treatments with log_10_ distance from cage, overlaid on raw data. Dashed lines indicate 95% confidence intervals of model fits. Grey dashed line indicates no change in weight, and negative values indicate kelp gained mass.

*Pycnopodia* caused a displacement in grazing away from the cage (figure 3, table 3), with a per cent decline in grazing of 73.4%, 60.3%, 6.5% and 49.1% at 0.25, 0.5, 0.75 and 1 m distance from the cage when *Pycnopodia* were present versus absent, respectively. At the array scale, sea stars reduced the amount of kelp grazed (table 3), with a 7.7% decrease in per cent kelp grazed relative to the control plots. Indeed, grazing after 24 h increased further from the cage when sea stars were present by 42.5%, 28.2% and 9.7% at 2, 2.75 and 3.75 m, respectively.

**Table 3.** Results of a general linear mixed effects model testing the fixed effects of sea star treatment, log_10_ *distance* from cage and their interaction on the proportion of kelp grazed by herbivores after 24 h. The model included *site*, *block* (nested within site) and *transect* (nested within *site*, *block* and *treatment*) as random factors.

effect	Sum Sq	Mean Sq	Num d.f.	Den d.f.	*F* value	Pr(>*F*)
treatment	0.51	0.51	1	216.8	5.9	0.016
log distance	11.55	11.55	1	381	134.3	<0.0001
treatment : log distance	0.35	0.35	1	381	4.1	0.044

#### (iii) Size of halo

**Figure 4.**
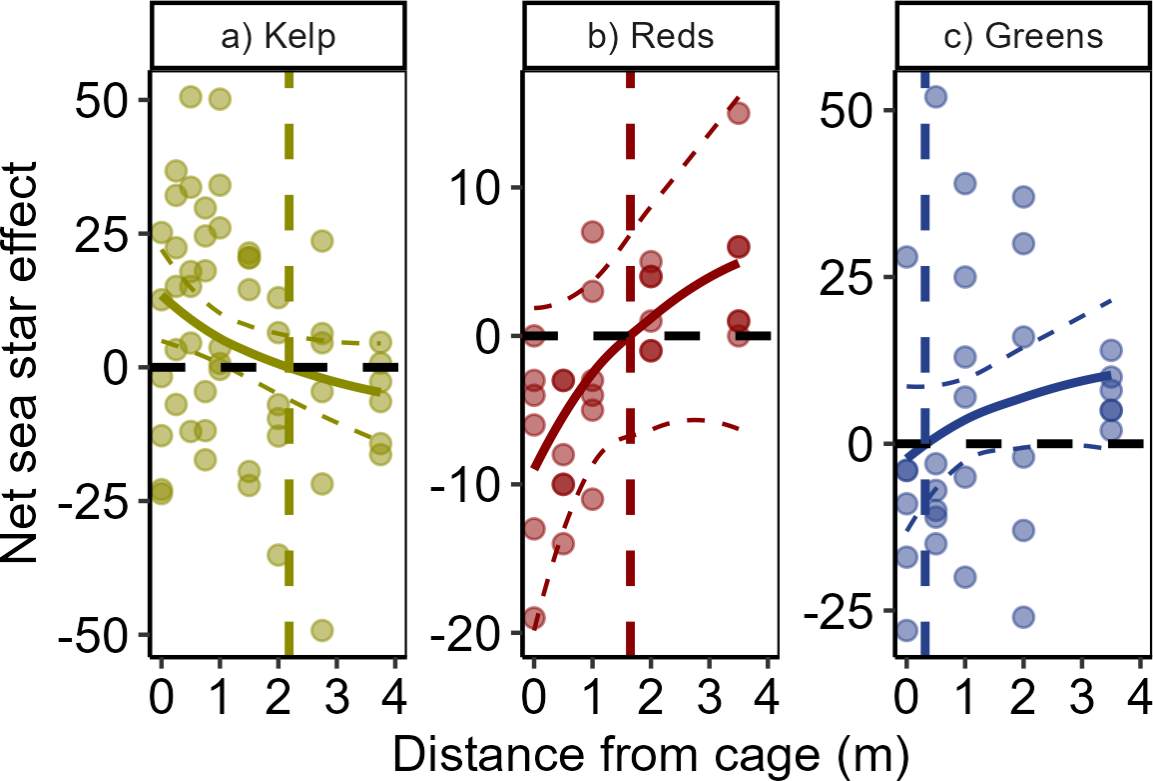
Net effects of *Pycnopodia* presence (versus absence) on the abundance of kelp, red sea urchins and green sea urchins at 24 h, which were measured as the average difference between paired sea star treatment and control plots at a given untransformed distance from the cage for difference in per cent kelp remaining (yellow) or red (red) or green (blue) urchin density per m^2^. The fit of the general log_10_-linear model is overlaid on raw data. Coloured vertical dashed lines indicate the size of the ‘halo’, i.e. the distance from the cage at which *Pycnopodia* had a net zero effect on each taxon (2.18, 1.65 and 0.32 m for kelp, red urchins and green urchins, respectively). Red and green urchins were *M. franciscanus* and *S. droebachiensis*).

Using a different analytical approach that averaged densities and changes in kelp mass across transects and compared the net effect of the sea star (see §2), we found the presence of an inactive, caged sea star suppressed red and green urchin densities closer to the cage and benefitted kelp (figure 4, table 4). By setting the net sea star effect to zero and solving each linear equation, we calculated the radius and area of the halo of influence effect (see §2). The halo effects on kelp and red urchins were very similar in size, with radii of 2.18 and 1.65 m and areas of 15.0 and 8.5 m^2^, for kelp and red sea urchins, respectively. The halo for green urchins was negligible at 0.32 m radius and 0.31 m^2^.

**Table 4.** Results of a general linear model testing the net effects of *P. helianthoides* sea star presence (measured as difference (paired sea star treatment – control plots at 24 h)) in per cent kelp remaining or urchin density per m^2^ varied with log_10_ *distance* from cage, species (kelp, red or green urchin) and their interaction.

effect	Sum Sq	d.f.	*F* value	Pr(>*F*)
LogDist_m	0.74	1	0	0.961
species	984.37	2	1.61	0.204
LogDist_m:Species	3052.10	2	4.99	0.008
residuals	33000.78	108		

## 4. Discussion

In a natural degraded habitat, we show that the presence of *Pycnopodia* affected the behaviour of red sea urchins in the field and caused a decrease in grazing within a >2 m radius and >15 m^2^ seafloor area for 24 h. While both red sea urchins (*M. franciscanus*) and green sea urchins (*S. droebachiensis*) were abundant in the study, only red sea urchins were deterred by *Pycnopodia*, and the spatial scale of their response aligned very closely with the reduced grazing effects on kelp. The spatial alignment between the effect of *Pycnopodia* on red sea urchins and on kelp consumption, despite little evidence of an effect on green sea urchins, supports previous interpretations that *M. franciscanus* is the dominant grazer in this system [25]. The importance of red sea urchins in controlling kelp biomass in this region has been found elsewhere [16,25] and was likely due to their larger body size relative to green sea urchins, even though green sea urchins were more abundant (table 1). This result is intriguing given previous research has suggested a size refuge for larger-bodied red sea urchins from *Pycnopodia*. Although the mean size of the red sea urchins was below the threshold suggested for the size refuge for red sea urchins (<8 cm diameter [15]), some ‘large’ red urchins were still present at our sites. Despite the variability in species response, our data demonstrate that *Pycnopodia* can have ecologically relevant indirect benefits to kelp biomass in degraded urchin barrens in the wild.

The ‘halo’ of displacement of red sea urchins surrounding the sea star occurred with a stationary sea star that was not able to touch nor actively pursue the sea urchins. This suggests that a waterborne chemical cue was the mechanism for the halo effect observed here [36,37,39,40,42,48]. Importantly, this waterborne chemical cue continued to elicit an 8.5 m^2^ halo response by red sea urchins for at least 24 h despite the presence of kelp and natural levels of ocean current, turbulence and diffusion of the cue in the surrounding seawater [40,48,49]. It should be noted that the *Pycnopodia* were fed red sea urchins during their acclimation in the laboratory, which could have affected *Pycnopodia*’s waterborne cues and therefore the cue responses of both red and green sea urchins [50]. It is also possible that some of the urchin responses and grazing effects we observed were not actually direct responses to *Pycnopodia* waterborne cues, but rather a consequence of urchin–urchin chemosensory cues [51–54].

While we observed a clear effect of the sea star on nearby red urchin density and subsequent benefits to kelp, there was a modest effect of *Pycnopodia* on the overall grazing rate in the whole approximately 50 m^2^ experimental area (7.7% reduction, table 3, figure 3). Rather, grazing was largely displaced from closest to the cage to further away from the sea star, with higher grazing at 2.75 and 3.75 m from the caged sea star than in the controls (figure 3), presumably due to the movement of red sea urchin individuals away from the sea star (figure 2). This suggests that a single, stationary *Pycnopodia* may primarily displace grazing pressure. It is possible that an uncaged, roaming sea star could elicit different fear responses among both red and green sea urchins, as has been reported elsewhere [39,52]. Thus, future research should address how the activity level of the sea star or multiple stationary *Pycnopodia* in close proximity to one another might affect community-level grazing pressure at larger scales.

Our data suggest that the kelp additions themselves attracted sea urchins from outside the experimental plots, and we expect that by deploying kelp and by situating our study in an urchin barren habitat, we may be underestimating the potential fear responses by both green and red sea urchins in kelp forests. For example, one day after kelp additions, the density of red and green sea urchins increased by an average of 193% and 303% in the control plots and by 87% and 285% in the sea star present plots, respectively. Increased hunger levels have been shown to diminish and even eliminate the fear responses of purple sea urchin congeners (*S. purpuratus*) to *Pycnopodia* [40]. Thus, we hypothesize that the lack of response of green sea urchins to the sea stars may have been influenced by kelp presence, their hunger level or both. Thus, continued research is also needed to investigate how kelp availability and hunger level interact with sea star cues to alter prey decision-making, behaviour and foraging rate for different urchin species.

Ultimately, our findings show ecologically relevant fear responses and reduced grazing rates by presumably hungry, gonad-deficient red sea urchins exposed to *Pycnopodia* cues. This occurred in urchin barren habitat in the wild despite the presence of good foraging opportunities (i.e. the experimentally provided kelp), without direct contact with the sea star, and despite a negligible response of green sea urchins. This is an important step demonstrating that non-consumptive effects of *Pycnopodia* may benefit kelp in degraded habitats in the wild, and our results likely underestimate the potential non-consumptive effects of the sea star in forested habitats. Regarding the role of *Pycnopodia* in kelp forest restoration and recovery efforts, many important questions remain. For example, the densities at our sites were lower than in some other urchin barrens, and sea urchin density has been shown to influence their trait-mediated responses to predators [41]. It is important to note that purple sea urchins (*S. purpuratus*) are the dominant sea urchin species preventing recovery in many areas of recent kelp collapse in the northeastern Pacific (but see [16]). The demonstrated effects on red sea urchins, as well as the negligible response of green sea urchins demonstrated herein, underscore the need for similar research examining the effects of *Pycnopodia* on purple sea urchins for greater inference in many areas currently experiencing kelp collapse.

## Data Availability

The data supporting this publication are housed at BCO-DMO. The data used for the site characterization can be found at [55]. The data used for urchin densities during the experiments can be found at [56]. The data used for kelp wet weights during the experiments can be found at [57]. Code used for analyses is included as electronic supplementary material. Supplementary material is available online [58].
